# Emergence of the First Strains of SARS-CoV-2 Lineage B.1.1.7 in Romania: Genomic Analysis

**DOI:** 10.2196/28049

**Published:** 2021-08-13

**Authors:** Andrei Lobiuc, Mihai Dimian, Olga Sturdza, Roxana Filip, Mihai Covasa

**Affiliations:** 1 Department of Human Health and Development College of Physical Exercise and Sport Stefan cel Mare University of Suceava Suceava Romania; 2 Department of Computers, Electronics and Automation College of Electrical Engineering and Computer Science Stefan cel Mare University of Suceava Suceava Romania; 3 Integrated Center for Research, Development and Innovation in Advanced Materials, Nanotechnologies, and Distributed Systems for Fabrication and Control Stefan cel Mare University of Suceava Suceava Romania; 4 Suceava County Emergency Hospital Suceava Romania

**Keywords:** infectious disease, COVID-19, strain, virus, Romania, transmission, spread, mutation, impact, case study, genome, sequencing, genetics, epidemiology, variant, virology, lineage

## Abstract

**Background:**

The United Kingdom reported the emergence of a new and highly transmissible SARS-CoV-2 variant (B.1.1.7) that rapidly spread to other countries. The impact of this new mutation—which occurs in the S protein—on infectivity, virulence, and current vaccine effectiveness is still under evaluation.

**Objective:**

The aim of this study is to sequence SARS-CoV-2 samples of cases in Romania to detect the B.1.1.7 variant and compare these samples with sequences submitted to GISAID.

**Methods:**

SARS-CoV-2 samples were sequenced and amino acid substitution analysis was performed using the CoV-GLUE platform.

**Results:**

We have identified the first cases of the B.1.1.7 variant in samples collected from Romanian patients, of which one was traced to the region of the United Kingdom where the new variant was originally sequenced. Mutations in nonstructural protein 3 (Nsp3; N844S and D455N) and ORF3a (L15F) were also detected, indicating common ancestry with UK strains as well as remote connections with strains from Nagasaki, Japan.

**Conclusions:**

These results indicate, for the first time, the presence and characteristics of the new variant B.1.1.7 in Romania and underscore the need for increased genomic sequencing in patients with confirmed COVID-19.

## Introduction

A new SARS-CoV-2 variant, with an N-Y substitution in the 501 position of the spike (S) protein, was detected in the United Kingdom in the fall of 2020. An initial variant of the virus, termed 501 N, with fewer mutations, occurred in late September in Wales, followed by the current variant (VUI-202012/01), giving rise to lineage B.1.1.7, which began to spread rapidly in the United Kingdom and then globally [1]. The new variant has 18 particular mutations, of which several have biological significance and are of epidemiological interest. Among the most notable mutations is N501Y, within the S protein, which corresponds to the receptor binding domain of the virus, where attachment to the host ACE2 enzyme takes place. Other important mutations are the deletion of two amino acids, histidine and valine, at positions 69 and 70, and a substitution at position 681, within the same spike protein. Of great concern is the increased transmissibility and disease severity compared to older variants, raising questions concerning its potential avoidance of successful nucleic acid amplification for diagnostic tests or even reduced vaccine effectiveness [2]. On January 8, 2021, Romania confirmed the first case of COVID-19 infection with the new strain, in a patient from Giurgiu (in South-East Romania) without a history of travel to the United Kingdom or contact with individuals from the United Kingdom. On January 22, 2021, two additional individuals from Bucharest were identified to have the new strain. They reported no travel history, were in good clinical condition, and were isolated at home under the supervision of a family physician. A fourth case was reported in Suceava County, in North-East Romania, on January 25, 2021, in an individual who arrived from the United Kingdom. A fifth reported case was confirmed on January 26, 2021, in a patient from Constanta, South-East Romania, with no travel history or contact with individuals infected with the new strain. Considering when B.1.1.7 was identified in Europe, its faster transmission compared to earlier strains, and the lack of genomic sequencing in Romania, there exists the possibility that the new variant is far more widespread in Romania than confirmed. In this paper, we report the identification of the new B.1.1.7 SARS-CoV-2 variant in Romania and present its characteristics in sequenced genome samples with the aim of enabling further comparison of transmission.

## Methods

### Overview

A total of 20 samples, collected from patients in the cities of Cluj and Craiova and Suceava County in Romania were selected for analysis, including patients with possible contacts with infected individuals from the United Kingdom. Sample viral titers and RNA amounts were quantified using quantitative polymerase chain reaction (qPCR) and Qubit fluorometers (Thermo Fisher Scientific), respectively. RNA extracts were reverse transcribed and libraries were prepared using AmpliSeq (Thermo Fisher Scientific) SARS-CoV-2 primer panels and workflow. Automatic library templating was performed using Ion Chef equipment and sequencing was carried out on Ion GeneStudio S5 with Ion 540 chips. Sequencing reads and assemblies were checked for quality using Ion Torrent Suite software plugins. Amino acid substitution analysis was performed using the CoV-GLUE platform. The B.1.1.7 SARS-CoV-2 sequence was uploaded into GISAID, under the ID EPI_ISL_869241. The consensus sequence and available Romanian sequences (from different laboratories) belonging to clade B.1.1.7 in Romania were aligned in GISAID to the reference strain using the MAFFT algorithm and maximum likelihood trees were obtained with MegaX software.

### Ethics Approval and Consent to Participate

The study was approved by the ethics committee of University Stefan cel Mare of Suceava, Romania (protocol 11733/14.07.2020) and of Suceava County Emergency Hospital (protocol 17669/13.07.2020). All participants provided individual informed consent.

## Results and Discussion

**Figure 1:**
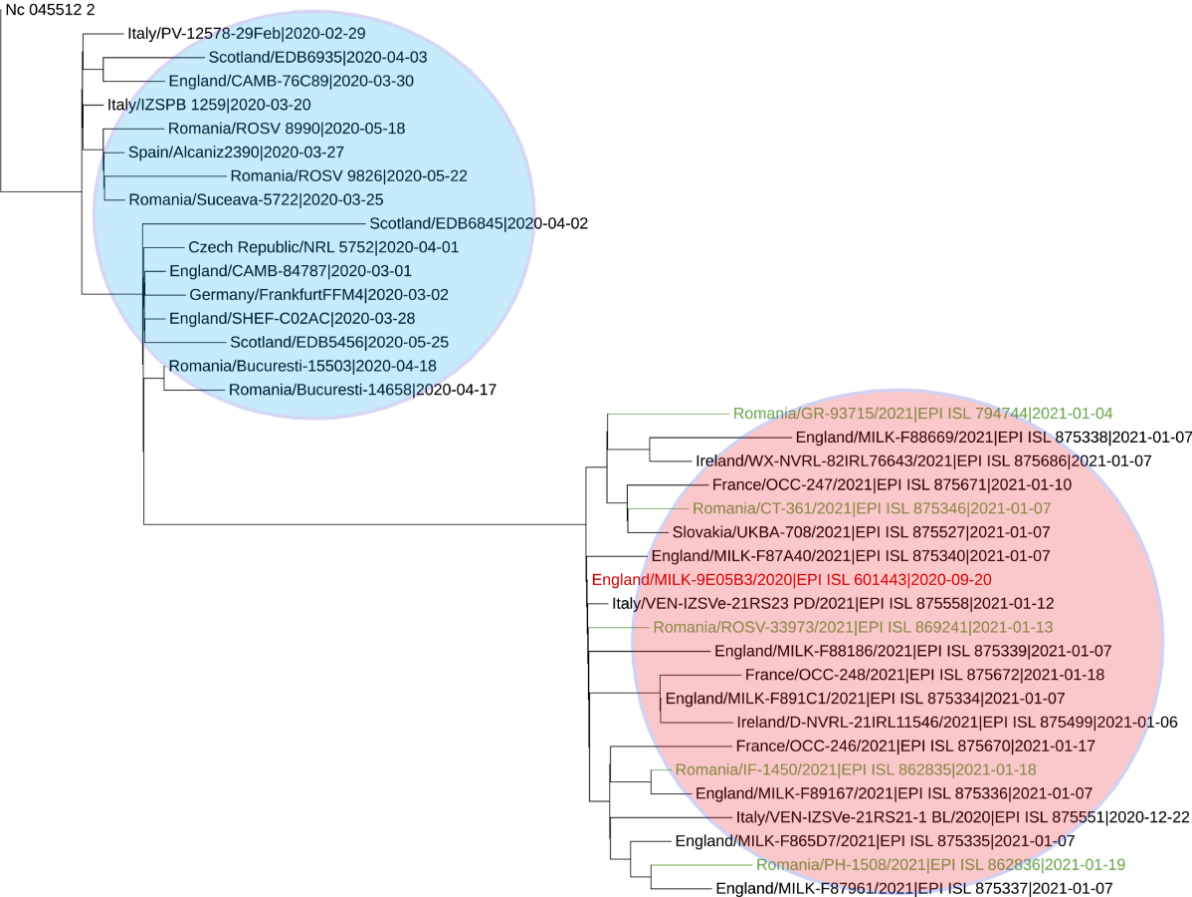
Phylogenetic placement of pre-B.1.1.7 samples (blue area) and B.1.1.7 samples (red area) from different European countries, including Romanian strains (green text).

Among the 20 samples sequenced by our laboratory, one presented characteristic mutations of the B.1.1.7 SARS-CoV-2 variant. Phylogenetic placement of this sample, as well as others from Romania within the same lineage included in GISAID, shows the clear distinction of this lineage from the early 2020 strains, including the ones from England and Wales (Figure 1).

A synopsis of all mutations found in all Romanian GISAID entries belonging to this clade was constructed (Multimedia Appendix 1). All Romanian samples share all 18 mutations characteristic of the B.1.1.7 strain; however, some of them have additional ones.

One such mutation is present only in the sample originating from Suceava, affecting the ORF8 protein, where a stop codon is gained by changing a C nucleotide to a T nucleotide in position 27,945 in the genome. According to CoV-Glue, this mutation has already been encountered in over 580 samples from April to October 2020. Of these, 73% (n=313) belong to specimens collected in the United Kingdom [3]. A second ORF8 truncation, not currently described for B.1.1.7 strains, appears in the samples from Giurgiu and Constanta, in position 68, also gaining a stop codon. Previous occurrences of this mutation are seen in 279 samples from CoV-Glue, of which 91% (n=256) are from the United Kingdom and 27% (n=76) originate from Milton Keynes laboratories, where the original B.1.1.7 strain was sequenced [4]. Such mutations indicate that, although B.1.1.7 originates in the United Kingdom, the set of characteristic viral alterations appeared much earlier and was grafted onto several different already circulating strains in the region. This idea is supported by the fact that, although the first sequenced samples carrying the new strain originated in Kent and Greater London, on September 20 and 21, 2020, respectively [5], the hallmark N501Y mutation first appeared in Italy in August 2020 [6]. However, at this point, the Romanian strains bearing the particular ORF8 mutations described above clearly originated in the United Kingdom, which is also supported by the fact that the patient from Suceava County resides in the United Kingdom and arrived in Romania shortly before the sample was sequenced. One other patient (EPI_ISL_794744) had no history of recent travel abroad but lived in a small city with a high number of individuals working abroad, including in the United Kingdom [7]. The remaining three patients from whom samples were sequenced had no travel history abroad or data were not available.

Strains without a functional ORF8 protein are considered to have epitope loss, which may decrease the accuracy of serological testing, whereas ORF8 antibodies could offer information on both acute and convalescent antibody response. Furthermore, ORF8 truncated proteins decrease disease severity and asymptomatic or mild cases might not be detected [8]. As such, the significance of ORF8 truncations in the context of B.1.1.7 strains should be promptly investigated, considering that mutations in the S gene characteristic to this lineage, particularly the deletion at positions 69-70, may elude detection by polymerase chain reaction (PCR) with certain diagnostic kits that have been used in the United Kingdom for a while [9]. This type of behavior could be indirectly but significantly linked to increased transmissibility of the virus, as potentially infected individuals might not have been accurately identified as such.

Another noteworthy mutation is N844S within nonstructural protein 3 (Nsp3) present in the Suceava sample, which is recorded in only 8 other samples sequenced so far, most of them also from England [10]. The sample from Prahova also has a mutation in Nsp3 (D455N), which has been recorded in only one other sample, collected in Japan [11] in April 2020, belonging to clade B1.1. The Prahova sample is again distinct from others in Romania through the appearance of L15F in ORF3a, a mutation recorded in 5 samples from Nagasaki, Japan, sampled in April 2020, among 243 samples collected worldwide, mostly from the United Kingdom [12]. Although the Japanese samples do not belong to the B.1.1.7 lineage, the coincidental presence of these mutations might indicate common ancestry with the Prahova sample. Other individual mutations in the Giurgiu and Ilfov samples are commonly observed in sampled UK strains. The Constanta sample displays two additional mutations not encountered in other Romanian samples. The first, in Nsp2, is a change from A to V in position 306, a mutation seen in other 209 GISAID samples. These samples were collected in the United Kingdom, Norway, Denmark, the United States, and Belgium [13]. The second mutation is in Nsp12 and is a change from K to N in position 160, which has been encountered in other 27 samples, including ones from the United States, Italy, and Scotland [14].

At the moment, there are over 32,500 B.1.1.7 accessions deposited in GISAID, out of which approximately 30,000 are from the United Kingdom and 5 are from Romania. This lineage is of major interest, due to the fact that three of its mutations might contribute to higher infectivity and transmissibility. Namely, the N50Y mutation of the S gene significantly increases its interaction force and number of interactions with the human receptor ACE2 [15,16]. The deletion of two amino acids at positions 69 and 70 in the same S gene leads to systematically biased diagnostic tests and doubles the reproductive advantage of the virus and viral particle numbers [17]. Furthermore, the P681H mutation of the S protein might influence the cleavage of the S protein due to its proximity to the S1/S2 furin cleavage site [18]. Identification of new mutations is crucial for designing diagnostic reagents [19], slowing transmission, and reconfiguring vaccines against new variants. In addition, particular mutations, besides those specific to B.1.1.7, may in the future aid in tracing virus movements across Romania and worldwide. The genomic data obtained by various laboratories throughout the country, including ours, are centralized by the National Centre for Surveillance and Prevention of Communicable Diseases, and transmitted to national and regional departments of public health. This, together with epidemiological data, helped public health officials to institute quarantine measures and other restrictions to control the transmission and spread of the virus.

However, many European countries, including Romania, lag in genomic sequencing and the European Union recommends increased focused sequencing based on epidemiological data, transmission rates, infectivity, treatment failure, and S-gene dropout in PCR testing. Several factors affected the timely acquisition of genome sequence data in Romania, such as a relatively small number of genomic laboratories in the country, the high costs associated with equipment and analyses, and a lack of specialized laboratory personnel. However, a thorough characterization of strains circulating in Romania is required, as it contributes to developing usable diagnostic tests and vaccines, especially in light of notable differences between strains belonging to the same clade and the evolutionary capacity of SARS-CoV-2.

## Appendix

Multimedia Appendix 1 Mutation analysis.

## Abbreviations

Nsp: nonstructural protein
PCR: polymerase chain reaction
qPCR: quantitative polymerase chain reaction
S: spike
