# Supplementary material for: Emergence of the First Strains of SARS-CoV-2 Lineage B.1.1.7 in Romania: Genomic Analysis
Source: JMIRx Med. 2021 Aug 13;2(3):e28049. doi: 10.2196/28049 (PMC8363123; doi:10.2196/28049)
Supplement: Multimedia Appendix 1 [file xmed_v2i3e28049_app1.docx]

**Multimedia Appendix 1**. List of mutations encountered in Romanian B.1.1.7 samples (green entries indicate mutations characteristic of B.1.1.7, while bold entries indicate singular non-B.1.1.7 mutations among Romanian samples).

| Genome region and original amino acid | | Position | Substituted amino acid | EPI_ISL_869241 (Suceava) | EPI_ISL_794744 (Giurgiu) | EPI_ISL_862835 (Ilfov) | EPI_ISL_862836 (Prahova) | EPI_ISL_875346 (Constanta) |
| --- | --- | --- | --- | --- | --- | --- | --- | --- |
| S | | | | | | | | |
|  | N | 501 | Y | Yes | Yes | Yes | Yes | Yes |
|  | A | 570 | D | Yes | Yes | Yes | Yes | Yes |
|  | D | 614 | G | Yes | Yes | Yes | Yes | Yes |
|  | P | 681 | H | Yes | Yes | Yes | Yes | Yes |
|  | T | 716 | I | Yes | Yes | Yes | Yes | Yes |
|  | S | 982 | A | Yes | Yes | Yes | Yes | Yes |
|  | D | 1118 | H | Yes | Yes | Yes | Yes | Yes |
|  | HV | 69-70 | Deletion | Yes | Yes | Yes | Yes | Yes |
|  | Y | 144 | Deletion | Yes | Yes | Yes | Yes | Yes |
| N | | | | | | | | |
|  | D | 3 | L | Yes | Yes | Yes | Yes | Yes |
|  | R | 203 | K | Yes | Yes | Yes | Yes | Yes |
|  | G | 204 | R | Yes | Yes | Yes | Yes | Yes |
|  | S | 235 | F | Yes | Yes | Yes | Yes | Yes |
| Nsp2 (ORF1ab) | | | | | | | | |
|  | A | 306 (486) | V |  |  |  |  | **Yes** |
| Nsp3 (ORF1ab) | | | | | | | | |
|  | T | 183 (1001) | I | Yes | Yes | Yes | Yes | Yes |
|  | N | 844 (1662) | S | **Yes** |  |  |  |  |
|  | A | 890 (1708) | D | Yes | Yes | Yes | Yes | Yes |
|  | I | 1412 (2230) | T | Yes | Yes | Yes | Yes | Yes |
|  | D | 455 | N |  |  |  | **Yes** |  |
| Nsp4 (ORF1ab) | | | | | | | | |
|  | F | 17 (2780) | L |  |  |  | **Yes** |  |
| Nsp6 (ORF1ab) | | | | | | | | |
|  | SF | 106-108 (3675-3677) | Deletion | Yes | Yes | Yes | Yes | Yes |
| Nsp12 (ORF1ab) | | | | | | | | |
|  | K | 160 (4552) | N |  |  |  |  | **Yes** |
|  | P | 323 (4715) | L | Yes | Yes | Yes | Yes | Yes |
| Nsp13 (ORF1ab) | | | | | | | | |
|  | K | 460 (5748) | R |  |  | **Yes** | **Yes** |  |
| Nsp14 (ORF1ab) | | | | | | | | |
|  | A | 119 (6044) | V |  | **Yes** |  |  |  |
|  | E | 347 (6272) | G |  |  | **Yes** |  |  |
| Nsp15 (ORF1ab) | | | | | | | | |
|  | P | 111 (6563) | T |  | **Yes** |  |  |  |
| ORF3a | | | | | | | | |
|  | L | 15 | F |  |  |  | **Yes** |  |
| ORF7a | | | | | | | | |
|  | Y | 20 | N |  |  |  | **Yes** |  |
| ORF8 | | | | | | | | |
|  | Q | 18 | Stop | **Yes** |  |  |  |  |
|  | Q | 27 | Stop | Yes | Yes | Yes | Yes | Yes |
|  | R | 52 | I | Yes | Yes | Yes | Yes | Yes |
|  | Y | 73 | C | Yes | Yes | Yes | Yes | Yes |
|  | K | 68 | Stop |  | Yes |  |  | Yes |
| Date of sample collection (MM/DD/YYYY) | | | | 01/13/2021 | 01/04/2021 | 01/18/2021 | 01/19/2021 | 01/07/2021 |
